# Riboswitch identification using Ligase-Assisted Selection for the Enrichment of Responsive Ribozymes (LigASERR)

**DOI:** 10.1093/synbio/ysz019

**Published:** 2019-07-08

**Authors:** Matthew C Haines, Marko Storch, Diego A Oyarzún, Guy-Bart Stan, Geoff S Baldwin

**Affiliations:** 1 Department of Life Sciences, Imperial College London, London, UK; 2 Imperial College Centre for Synthetic Biology, Imperial College London, London, UK; 3 London BioFoundry, Imperial College Translation & Innovation Hub, London, UK; 4 School of Informatics, University of Edinburgh, Edinburgh, UK; 5 School of Biological Sciences, University of Edinburgh, Edinburgh, UK; 6 Department of Bioengineering, Imperial College London, London, UK

**Keywords:** RNA, ribozyme, riboswitch, synthetic biology, gene circuit engineering

## Abstract

*In vitro* selection of ligand-responsive ribozymes can identify rare, functional sequences from large libraries. While powerful, key caveats of this approach include lengthy and demanding experimental workflows; unpredictable experimental outcomes and unknown functionality of enriched sequences *in vivo*. To address the first of these limitations, we developed Ligase-Assisted Selection for the Enrichment of Responsive Ribozymes (LigASERR). LigASERR is scalable, amenable to automation and requires less time to implement compared to alternative methods. To improve the predictability of experiments, we modeled the underlying selection process, predicting experimental outcomes based on sequence and population parameters. We applied this new methodology and model to the enrichment of a known, *in vitro*-selected sequence from a bespoke library. Prior to implementing selection, conditions were optimized and target sequence dynamics accurately predicted for the majority of the experiment. In addition to enriching the target sequence, we identified two new, theophylline-activated ribozymes. Notably, all three sequences yielded riboswitches functional in *Escherichia coli,* suggesting LigASERR and similar *in vitro* selection methods can be utilized for generating functional riboswitches in this organism.

## Introduction

Biology uses an array of sensory elements to respond to changes in the environment. This includes riboswitches derived from RNA (1). Certain riboswitches rely on the self-cleaving property of ribozymes when altering gene expression (2, 3). For these ribozyme-based riboswitches, ribozyme activity is dependent on ligand concentration, with cleavage altering RNA sequence and hence function.

In addition to natural examples, there are several instances where synthetic ribozyme-based riboswitches have been developed (4–8).These synthetic elements have previously been generated rationally (4) or by using selection or screening methods that function *in vitro* (5, 6) or *in vivo* (7, 8). While all of these strategies were successful at some level, each suffers from certain limitations. For instance, rational methods are limited by their ability to predict RNA tertiary structures from sequence (9). Such interactions are often required for riboswitch function (10). *In vivo* methods meanwhile require control over intracellular ligand concentrations. For toxic ligands or for those that are unable to diffuse across the plasma membrane, control of intracellular conditions becomes problematic (11), limiting their application.

In contrast, *in vitro* methods avoid the presence of cellular membranes, facilitating easier control over ligand concentrations. Furthermore, 10^14^–10^15^ variants are commonly sampled when using *in vitro* selection methods (12). Due to the cell transformation bottleneck (13), this is at least four orders of magnitude larger compared to *in vivo* methods, enabling the exploration of larger sequence spaces. That said, *in vitro* selection methods have several important limitations. Firstly, this process requires a large investment of time and human resources compared to alternative rational (4) or lower-scale screening methods (6). Secondly, there are few tools available to predict the outcome of selection experiments and to optimize parameters, e.g. selection stringencies. Thirdly and perhaps most importantly, *in vitro*-selected ribozymes do not always translate into functional riboswitches when applied intracellularly. For example, in two reports selected sequences have failed to yield riboswitches functional in mammalian cells (5, 14). Such results have created uncertainty regarding the ability of *in vitro* selection methods to yield sequences that are functional intracellularly (15).

To address the time and human resources required for selection, we set out to conceive an *in vitro* selection method which required less time and can be scaled in 96-well microtiter plates. Achieving the latter benchmark would increase the number of parallel selections, while acting as precursor to automation in subsequent experiments (16). To achieve these aims, we developed a novel method referred to as Ligase-Assisted Selection for the Enrichment of Responsive Ribozymes (LigASERR). During LigASERR, preferential ligation of cleaved or full-length variants provides the basis for selection through simple pull-down assays. We demonstrate the functionality of LigASERR by enriching a theophylline-activated sequence, characterized previously through *in vitro* selection (14). We enrich this functional sequence from a library designed to test the performance of key positive and negative selection steps. In addition to the development of LigASERR, we utilized mathematical tools to identify optimal selection conditions and to estimate the number of cycles required for sufficient target sequence enrichment. These tools can be applied to more challenging selection experiments, guiding the exploration of sequence space for functional sequences. As a final contribution, we show three of the enriched theophylline-activated ribozymes are able to function as riboswitches in *Escherichia coli*, suggesting LigASERR and similar *in vitro* methods can be reliably used for riboswitch generation.

## Materials and methods

### RNA secondary structure prediction

RNA secondary structures were calculated using Vienna RNAfold software (17) and interpreted using VARNA (18).

### DNA oligonucleotides

Chemically synthesized oligonucleotides were purchased from Integrated DNA Technologies, Inc. or Twist Bioscience and are available in the Supplementary Data.

### *In vitro* ribozyme assays

DNA templates were prepared from oligonucleotides using polymerase chain reaction (PCR) and Sense and Anti-sense primers. Amplified DNA was purified using GenElute™ PCR Clean-Up spin-columns (Sigma). *In vitro* transcription was conducted in 40 mM Tris-HCl (pH 7.9), 2 mM spermidine, 8.4 mM MgCl_2_, 2 mM each ribonucleotide triphosphate, 25 ng/μl DNA template, 1 U/μl New England Biolabs (NEB) RNase Inhibitor Murine; 5 mM dithiothreitol, 5 U/μl NEB T7 RNA Polymerase and varying theophylline (Sigma). Where required, RNA was purified using solid-phase reversible immobilization (SPRI) beads (Supplementary Data) and reverse transcribed using NEB Protoscript^®^ II RTase in the presence of 5 μM HEX-labeled RT primer. TRT reactions were otherwise assembled using the above *in vitro* transcription components except MgCl_2_ was present at a final concentration of 10.4 mM and the following components were added: 75 mM KCl, 0.5 mM each dNTP, 2.5 μM HEX-labeled RT primer and 10 U/μl NEB Protoscript^®^ II RTase. TRT reactions were incubated at 37°C, followed by inactivation at 65°C for 20 min. All cDNA was purified using SPRI beads (Supplementary Data) and RNA digested by incubating at 37°C for 30 min in the presence of 0.1 mg/ml RNase A. All samples analyzed via electrophoresis were denatured in 1× formamide loading buffer (47.5% deionized formamide, 0.0125% bromophenol blue, 0.0125% xylene cyanol, 0.0125% sodium dodecyl sulphate, 5 mM ethylenediaminetetraacetic acid (EDTA) and 50 mM NaCl) and separated on 8% polyacrylamide, 8 M urea, 1× Tris-borate-EDTA gels. RNA was stained using SYBR™ Green II (Thermo Fisher Scientific) and imaged using a Fujifilm LSA-3000 imager. HEX-labeled cDNA was imaged using a Fujifilm FLA 5000 imager (532 nm laser and 570DF20 filter). Band intensities were quantified using TOTALLAB CLIQS 1D Gel Image Analysis software. To calculate RNA molar amounts, intensities were normalized to their corresponding molecular weights.

### LigASERR selection

Biotinylated Control Library DNA was prepared from the corresponding oligonucleotide using semi-quantitative PCR (Supplementary Data) with Sense_bio and Anti-sense_bio primers. From this, cDNA was synthesized under selection conditions and biotinylated DNA removed by washing with Dynabeads™ MyOne™ Streptavidin C1 (Thermo Fisher Scientific) resuspended in the recommended B&W buffer but at a final concentration of 1.25 mg/ml. Three washes were conducted by transferring supernatants to additional aliquots of Dynabeads. cDNA was subsequently purified using SPRI beads and RNA digested as previously described. Purified cDNA was annealed to 1 μM Splint in the presence of 1 μM Adapter F or C under 1× NEB *Taq* DNA Ligase Reaction Buffer. On ice NEB *Taq* DNA Ligase was added to a final concentration of 1.6 U/μl and the reaction incubated at either 51.7°C or 46.4°C for 30 min to ligate full-length or cleaved cDNA, respectively. Ligation was quenched with 20 mM EDTA (final concentration) and ligated DNA immobilized and washed with NaOH using Dynabeads according to the manufacturer’s instructions. Selected cDNA was amplified with semi-quantitative PCR.

### Illumina sequencing and data analysis

DNA templates were prepared for sequencing in a similar manner to ‘16S Metagenomic Sequencing Library Preparation’ (Illumina). Both amplicon and indexing PCRs were implemented using NEB Phusion DNA polymerase. IllumSense and IllumAnti-sense primers were used during amplicon PCRs. Indexed DNA templates were pooled and sequenced using an Illumina MiSeq v2 150 PE micro run with a PhiX spike-in of 20%. Adapters were removed from demultiplexed reads using Cutadapt version 1.16 (19) and overlapping paired-end reads merged using PEAR v0.9.11 (20). Unique sequences in the entire dataset were identified using MATLAB R2018a and their frequency determined as a function of selection.

### *In vivo* assays

Each insert was assembled by incubating sfGFP_twist with an additional sequence acquired from Twist Bioscience, under BASIC linker ligation reaction conditions (21) in the presence of LMP-P and LMS-S linkers (Biolegio). Linker-ligated inserts were cloned into linker-ligated AmpR-pUC and transformed into *E. coli* DH5α. Resulting clones were incubated at 37°C overnight in LB media (ForMedium) supplemented with 50 μg/ml carbenicillin. Plasmid DNA was purified from cultures using E.Z.N.A.^®^ Plasmid Mini Kit I (Omega Bio-tek) and sequences verified by Source BioScience using SEVA_T0_rev and SEVA_T1_for primers. All plasmid sequences are available in the Supplementary Data.

Assembled expression cassettes were transformed into *E. coli* BL21(DE3) and picked colonies incubated in 200 μl LB medium supplemented with carbenicillin shaking overnight at 30°C. Overnight cultures were diluted 200 times into 100 μl LB supplemented with carbenicillin, 250 μM isopropyl β-D-1-thiogalactopyranoside and 0–2.5 mM theophylline. Cultures were grown shaking at 30°C to mid-log phase (6 h) and 2 μl off-sampled into 200 μl Phosphate Buffer Saline supplemented with 2 mg/ml kanamycin. Samples were analyzed for GFP fluorescence using a BD Fortessa flow cytometer and data analyzed using FlowJo_V10.

### Materials availability

All DNA constructs are available upon request following the completion of a Materials Transfer Agreement and any other required documentation.

## Results

### Methodology for ribozyme selection

**Figure 1.**
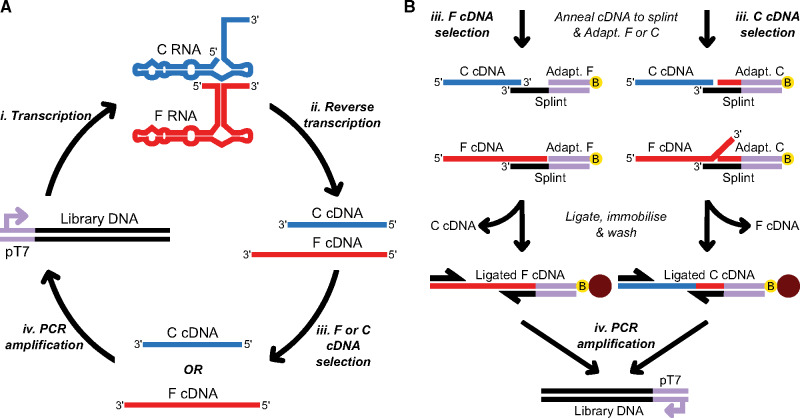
LigASERR scheme. (**A**) During a single selection, Library DNA is transcribed yielding cleaved (abbreviated C) and full-length (abbreviated F) RNA, respectively (step *i*). RNA is reverse transcribed yielding cDNA (step *ii*). Either C or F cDNA is selected depending on the phenotype being enriched (step *iii*). Selected cDNA is PCR amplified (step *iv*). (**B**) Selection of full-length or cleaved cDNA (step *iii*) is initiated by annealing cDNA to a Splint with either Adapter (Adapt.) F or C, respectively. Only the desired cDNA forms a ligase substrate. Selected cDNA is ligated, ensuring sequences required for DNA template regeneration are incorporated. Additionally, the 3′ biotin modification present on each adapter facilitates immobilization and subsequent washing on streptavidin-coated paramagnetic beads, preparing the pool for amplification.

Selection *in vitro* for ligand-responsive ribozymes is typically undertaken at the RNA level by separating cleaved and full-length variants using polyacrylamide gels (5, 14, 22). To overcome this labor-intensive approach, we implemented selection at the cDNA level. Given the stability of cDNA and its amenability to enzymatic modification, this opened up alternative methods of selection that are not possible otherwise. To select at the cDNA level we transcribed library DNA using T7 RNA polymerase and then perform reverse transcription, capturing ribozyme activity in a stable cDNA output (Figure 1A). This cDNA output is then fed into an enzymatically driven selection process, prior to PCR amplification of selected variants.cDNA selection is based on a ligase-mediated strategy (Figure 1B). The efficient ligation of a cDNA molecule to a second oligonucleotide can be achieved via annealing to partially double-stranded DNA composed of a longer splint and a shorter adapter oligonucleotide. The splint serves to provide a ligatable junction, while the overhang can be used to discriminate between cDNAs. The adapter oligonucleotide contains a 3′-biotin, facilitating downstream immobilization of the ligation reaction product. The use of Adapter C or F during cDNA annealing enables ligation to cleaved or full-length cDNAs, respectively. This is due to other cDNAs producing non-ligatable gaps or flaps (Figure 1B), since the *T*th DNA ligase has high fidelity for precisely matched DNA junctions (23, 24). Following ligation, the biotinylated ligation product is purified by immobilizing on streptavidin-coated paramagnetic beads and washing with NaOH to remove unligated material. Given the adapters contain the T7 promoter sequence and in the case of Adapter C, the sequence previously released during ribozyme cleavage, the intact DNA template is effectively regenerated during ligation, enabling straightforward amplification via PCR, completing the process of LigASERR.

### Design of a control library

**Figure 2.**
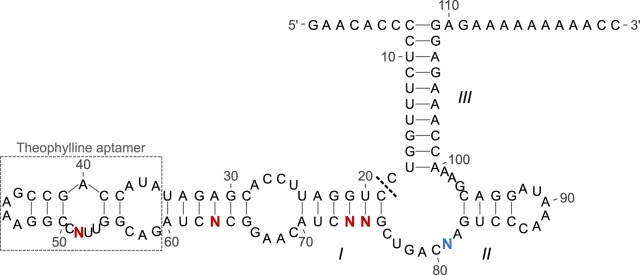
Sequence and structure of the Control Library. 5′ and 3′ termini are indicated along with the theophylline aptamer domain and stem numbers according to hammerhead ribozyme convention (25). The scissile bond is denoted by a dotted line. Degenerate bases incorporated to yield inactive and CA sequences are indicated by red and blue, respectively.

To test LigASERR functionality, we conducted a control experiment to enrich a known, ligand-activated ribozyme from a library of sequences. We based the design of the ligand-activated ribozyme on the theophylline-activated, VI-1 ribozyme sequence (14). We reasoned this ribozyme would function as an ideal test bed for our selection method given its large dynamic range and the extensive characterization of both its hammerhead ribozyme and theophylline aptamer domains. To increase its compatibility with our selection method, we modified stem III of the hammerhead ribozyme domain and regions distal to this region. These modifications should not compromise functionality given these sequences are not directly involved in catalysis or communication of ligand binding. The resulting Positive Control (PC) sequence formed the basis of the subsequent Control Library design (Table 1 and Figure 2).

**Table 1.** The genotype for each of the sequences listed is given at each degenerate loci within the Control Library (Figure 2)

	Locus identity				
Sequence(s)	52	63	73	74	81
Positive control (PC)	C	C	U	C	G
Inactive	N	N	N	N	H
Constitutively active (CA)	D	U	C	A	G

Nucleotide base identities follow the IUPAC nomenclature (26).

The VI-1 sequence was previously enriched through alternating positive and negative selection steps, generally required for the enrichment of ligand-responsive ribozymes. To demonstrate the ability of our methodology to generally select ligand-responsive ribozymes, we aimed to show positive and negative selections being implemented during the Control Experiment. To this end, we incorporated inactive and constitutively active (CA) sequences into the initial library. The successful enrichment of the PC would then be accompanied with a reduction in the frequency of inactive and CA sequences, demonstrating functional positive and negative selection steps, respectively (Supplementary Table S1).

To introduce inactive sequences into the Library, we diversified the PC at position 81. Lack of G at position 81 in combination with a defined C at position 76 significantly reduces rates of ribozyme catalysis (27). Therefore, ¾ of the starting Library would be inactive (Table 1). The remaining degenerate bases were incorporated to introduce CA sequences. These sequences are defined by fast cleavage rates, regardless of the theophylline concentration. We therefore diversified position 52, yielding sequences which could not be influenced by theophylline due to the absence of a C residue at this position (28). From this set, CA sequences were generated via degenerate base substitutions at positions 63, 73 and 74 (Table 1). This ensured a catalytically active conformation (Figure 2) could be formed without the stabilizing effect of theophylline binding. Note, this conformation contrasts with the predicted secondary structure of the PC in a theophylline-unbound conformation (Supplementary Figure S1). The resulting Control Library contained 1024 sequences, of which only the PC was a known theophylline-activated ribozyme.

### Optimization of selection conditions

Having designed the Control Library, we aimed to enrich the PC using as few cycles of selection as possible, where a selection cycle is defined as one positive selection followed by one negative selection. To aid the identification of optimal selection conditions, we derived a metric for the propensity of a sequence to be enriched. We refer to this metric as fitness and note that it is directly proportional to the rate of enrichment during selection (Supplementary Data, Equation (S15)). For the *i*th sequence, fitness ($\mathrm{fitnes}s_{i}$) under specific selection conditions is defined as:

(1) $$\mathrm{fitnes}s_{i}=r_{i}^{\left(+\right)}\left(1-r_{i}^{\left(-\right)}\right),$$

where $r_{i}^{\left(+\right)}$ and $r_{i}^{\left(-\right)}$ are the molar fractions of sequence i, cleaved under positive and negative selection conditions, respectively. For instance, $r_{i}^{\left(+\right)}=\frac{C_{i}^{\left(+\right)}}{C_{i}^{\left(+\right)}+F_{i}^{\left(+\right)}}$, where $C_{i}^{\left(+\right)}$ and $F_{i}^{\left(+\right)}$ are the molar amount of cleaved and full-length sequence i, respectively, measured under positive selection conditions. As outlined in the Supplementary Data, several assumptions were made in order to derive this expression.

To enrich the PC using a minimum number of cycles, the fitness of this sequence should be at a maximum relative to other sequences (Supplementary Data and Equation (S15)). From the Control Library design (Figure 2 and Table 1), the majority of sequences during the initial cycles of selection will either be inactive or CA. According to Equation (1) the fitness of these sequences is ∼0, regardless of the selection conditions. This is because either $r_{i}^{\left(+\right)}$ or $r_{i}^{\left(-\right)}$ are ∼0 or 1, respectively. From this, conditions that maximize the absolute fitness of the PC should maximize its rate of enrichment during the initial cycles.

**Figure 3.**
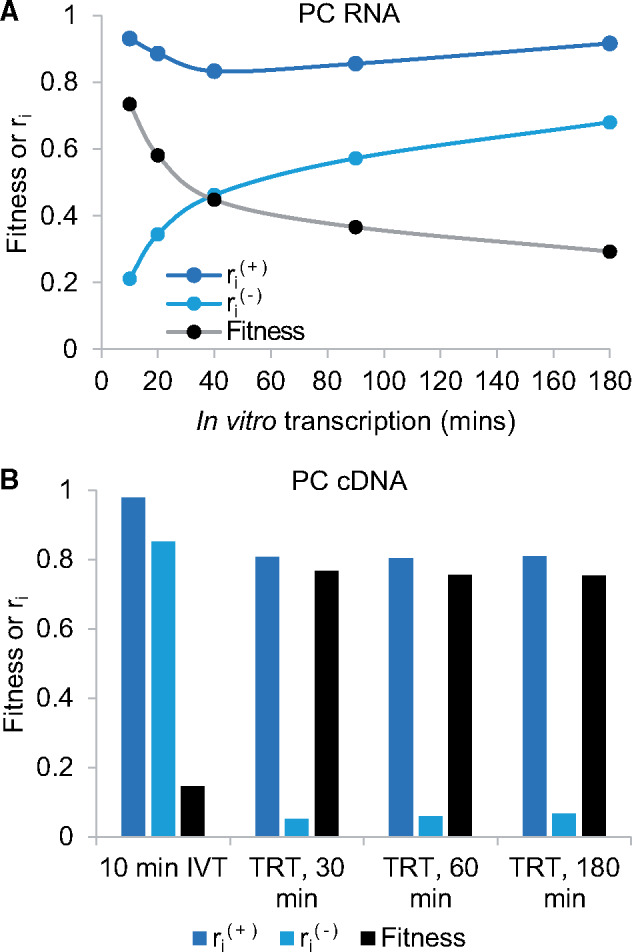
PC cleavage responses in the presence of 3.16 ($r_{i}^{\left(+\right)}$) or 0 ($r_{i}^{\left(-\right)}$) mM theophylline in addition to fitness values calculated according to Equation (1). (**A**) PC RNA responses and fitness calculated as a function of *in vitro* transcription (IVT) time. (**B**) PC cDNA responses and fitness quantified from reverse transcribed, purified RNA yielded from a 10-min IVT reaction or from cDNA synthesized for variable times under transcription, RT (TRT) reaction conditions. Representative data from typical reactions are shown, while the raw data are illustrated in Supplementary Figure S2.

Armed with these insights, we first investigated varying transcription times to optimize the fitness of synthesized PC RNA. To achieve this, PC DNA was *in vitro* transcribed for varying times under either 3.16 or 0 mM theophylline. These concentrations were chosen to represent positive and negative selection conditions, respectively, with 3.16 mM theophylline chosen as it yielded a maximum cleavage rate for the similar VI-1 sequence (14). The resulting transcription reactions were separated via electrophoresis and $r_{i}^{\left(+\right)}$ or $r_{i}^{\left(-\right)}$ determined (Figure 3A). These values were used to calculate fitness according to Equation (1) for each time point. Figure 3A illustrates that the fitness of the PC initially decreases rapidly with incremental transcription times. For instance, incubating this reaction for 20 min rather than 10 min, reduces PC fitness from 0.73 to 0.58; a reduction of ∼25%. Given cleavage in the presence of theophylline saturates quickly ($r_{i}^{\left(+\right)}$), this result is primarily driven by an increase in background cleavage in the absence of ligand over time ($r_{i}^{\left(-\right)}$). This suggested short transcription times were required to limit selection cycle number.

We subsequently analyzed the fitness of PC cDNA, generated following RT and prior to ligase-assisted selection. This was first achieved using a short, 10-min transcription reaction followed by RNA purification and subsequent RT. The resulting cDNA was separated via electrophoresis and fitness determined in an equivalent manner to Figure 3A (10 min IVT: Figure 3B). From this data, PC cDNA fitness reduced by 80% from 0.73 to 0.15 when compared to equivalent PC RNA fitness (Figure 3A). Analyzing $r_{i}$ values, this result was primarily driven by a significantly larger $r_{i}^{\left(-\right)}$ value suggestive of background cleavage during RNA purification and RT.

To overcome this, we conducted transcription and RT reactions simultaneously (TRT reaction). Under TRT reaction conditions we hypothesized partial reverse transcription would quickly attenuate ribozyme activity, reducing background cleavage. We incubated PC DNA under TRT reactions for 30, 60 or 180 min and measured $r_{i}^{\left(+\right)}$ or $r_{i}^{\left(-\right)}$ to calculate fitness (Figure 3B). Under these conditions, the fitness of the PC is significantly improved to a value of ∼0.75. A further observation is that the response of the PC is independent of incubation time. This is consistent with the view that a fixed time is required to attenuate ribozyme activity through partial reverse transcription. While other cDNA preparation methods were investigated (data not shown), the TRT reaction achieved the largest improvement in fitness and as such was used during the subsequent Control Experiment.

### Cycles required for PC enrichment

Prior to conducting the Control Experiment, we derived and implemented an algorithm to simulate the dynamics of the PC using fitness values from this sequence and the Control Library as arguments (Supplementary Data and Supplementary Figure S3). Such information could be used to determine the cycles and hence time required for PC enrichment. The derivation of expressions to implement the above algorithm involved the key assumption that all sequences except for the PC are diluted at the same rate during selection. While this is unlikely to be true for all selection experiments, this assumption should hold for the initial cycles of the Control Experiment. At this stage many sequences will be inactive or CA, having similar fitness values (∼0) and hence dynamics. The predicted dynamics of the PC suggested this sequence could be enriched to > 50% of the pool within five cycles of selection and close to 100% of the pool within eight cycles. This number of cycles was feasible given each selection could be manually implemented within 8 h (Supplementary Table S3) and as such the entire experiment completed within 2 weeks.

### Dynamics of sequences during selection

In line with our prediction from the previous section, the Control Library was subjected to five cycles of selection. During each cycle, positive selection was implemented before negative selection by synthesizing cDNA under TRT reaction conditions in the presence of 3.16 mM theophylline. Cleaved cDNA was selected, and the library PCR amplified. During negative selection, cDNA was similarly generated but in the absence of theophylline. Full-length cDNA was selected and the pool PCR amplified prior to further cycles. To monitor the enrichment of a theophylline-activated phenotype, the pool following each positive and negative selection was transcribed for 10 min under 3.16 or 0 mM theophylline (Supplementary Figure S4A). Additionally, we analyzed the cleavage response of the pool following five cycles and compared it to the response of the PC sequence (Supplementary Figure S4B). The similarity between the two responses suggested the PC or sequences with a similar phenotype had been significantly enriched.

We subsequently analyzed the Control Experiment at the single molecule level using Illumina sequencing (Materials and methods section). The resulting data would indicate whether sequences from the Control Library followed the expected dynamics and would enable a more detailed analysis. Using the above approach, we sampled 16 600 sequences from the initial pool, equivalent to 16-fold coverage of the Control Library. We sampled an equivalent number of sequences following each selection iteration (Supplementary Figure S5), allowing us to monitor the enrichment or dilution of sequences.

**Figure 4.**
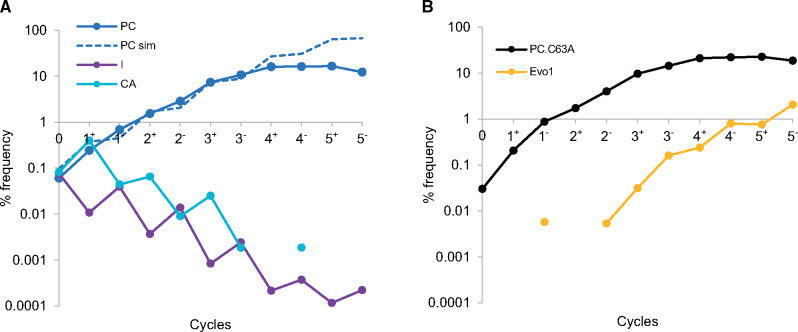
Dynamics of known and unknown sequences during selection. Each selection cycle is composed of an individual positive (+) and negative (−) selection. The absence of circles or line-segments indicates the relevant sequence or sequences were not recorded, yielding a % frequency of 0. (**A**) PC dynamics, mean dynamics of inactive (*n* = 768), abbreviated I and CA (*n *= 3) sequences, abbreviated CA, all from the Control Library along with simulated dynamics of the Positive Control (PC sim). (**B**) The dynamics of previously unknown PC.C63A and Evo1 sequences.

We first analyzed the dynamics of key Control Library sequences to confirm the functionality of the LigASERR method (Figure 4A). As expected the frequency of the PC increased as a function of selection cycles. Specifically, this sequence was enriched from 0.06 to a maximum of 16.6% following the fifth positive selection. Figure 4A also illustrates that there is a significant decrease in the frequency of both Control Library CA and inactive sequences. For instance, CA sequences were only recorded once following three cycles of selection, yielding an average frequency of 1:200 000 for this later portion of the experiment. Inactive sequences meanwhile decrease ∼1000-fold, from 1:1000 to 1:1 million. It is also interesting to note the changes in frequency for these sequences following individual positive and negative selections. For instance, while the frequency of CA sequences increases during positive selections, the opposite occurs for inactive sequences. The reverse is true during negative selection when the frequency of inactive sequences increases. These dynamics agree with the anticipated phenotypes of these sequences and the expectation that positive and negative selection were functional (Supplementary Table S1).

Figure 4A also illustrates that the dynamics of the PC, simulated using the model of section are accurate up to and including the third cycle (*R*^2^ = 0.97). Following the third cycle of selection, there appears to be deviation between actual and simulated dynamics. We hypothesized that this effect might be caused by the enrichment of other sequences, a scenario not accounted for under the assumptions of the simulation.

Following an analysis of the dynamics of the remaining sequences from the Control Library, we discovered that a sequence with a single C63A point mutation relative to the PC was similarly enriched to a maximum of 22.6% by the fifth positive selection. Furthermore, an additional sequence (Evo1) containing three substitution mutations relative to the PC (A68U, U73G and C74A) was detected after 1 cycle of selection. This sequence was then rapidly enriched to 2.1% of the pool by the end of selection (Figure 4B).

We determined predicted secondary structures for these new sequences, both without any constraints and with a constrained theophylline aptamer domain (Supplementary Figure S6). We conducted the latter to simulate the effect of theophylline binding on each sequence. Comparing these structures with those generated by the PC would highlight similarities and differences. For PC.C63A and PC structures, we note only minor differences between unconstrained structures and essentially no difference where the theophylline aptamer has been constrained. In contrast the Evo1 sequence yields significantly different structures, indicative of a different mechanism of function. We note that while the Evo1 sequence yielded a constrained structure similar to the active conformation, this was not the case for the other two sequences. This failure to predict an active conformation upon ligand binding is likely a result of the software (Materials and methods section), which does not consider effects such as stabilizing tertiary interactions nor the kinetics of transcription.

### Regulation of gene expression using selected ribozymes

Ligand-activated ribozymes have the potential to act as riboswitches for the control of gene expression (5, 6). We set out to determine if any of the sequences enriched during the Control Experiment could fulfill this function in *E. coli*. It was envisaged that each sequence would regulate GFP expression as previously described (7, 29), whereby RBS sequestering and RNA degradation is regulated by ribozyme activity. In addition to characterizing enriched sequences, we characterized a constitutively active (CA.A52) and an inactive variant of the PC (PC.G81A), both present in the Control Library. These sequences should strongly and weakly express GFP respectively, providing reference points.

**Figure 5.**
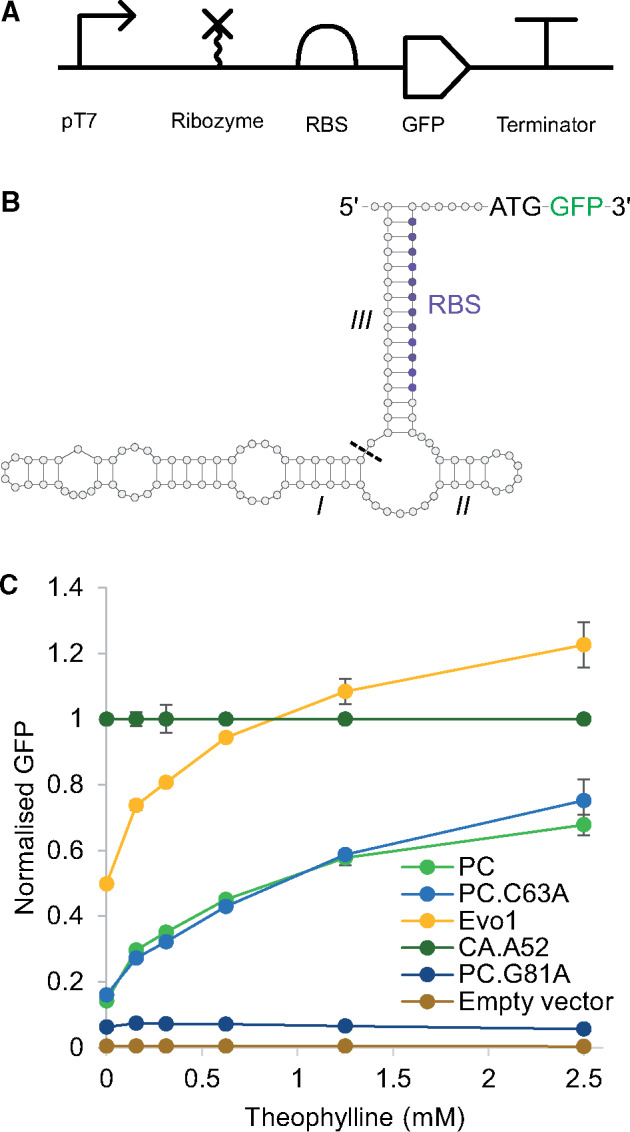
Ability of characterized ribozymes to regulate gene expression in *E. coli*. (**A**) SBOL (30) illustration of expression cassettes used to assay selected ribozymes and other Control Library sequences *in vivo*. (**B**) Transcribed mRNA. Hammerhead ribozyme stems are indicated along with the scissile bond (dotted line), RBS, GFP and 5′ and 3′ termini. (C) Cells harboring an empty vector or expression cassettes containing PC, PC.C63A, Evo1, CA.A52 or PC.G81A sequences were analyzed via flow cytometry under variable theophylline concentrations (0–2.5 mM). Geometric mean values were normalized to that of the CA.A52 sequence. Error bars denote standard error of the mean from two repeats.

To enable testing of sequences for riboswitch functionality, sequences were placed upstream of a *gfp* gene and under the control of the T7 promoter (Figure 5A). Stem III was also modified to encompass a complete RBS sequence, attenuating translation until cleavage and subsequent stem III dissociation (Figure 5B). Additionally, in this configuration mRNA stability should increase following ribozyme cleavage via the conversion of the 5′ triphosphate group to a hydroxyl (31), further enhancing GFP expression. Expression cassettes as illustrated in Figure 5A were cloned into high copy number plasmids using BASIC DNA assembly (21). Transformed cells containing constructs were subsequently grown under theophylline concentrations spanning 0–2.5 mM. The resulting GFP expression in individual cells was then quantified via flow cytometry. From this data, unimodal populations across all samples were observed (Supplementary Figure S7), implying geometric means could be used to compare responses.

Further analysis indicated that with respect to cells harboring the constitutively active CA.A52 sequence, those harboring PC, PC.C63A and Evo1 sequences all increased GFP expression in response to increasing theophylline (Figure 5C). Specifically, these sequences increased mean absolute GFP gene expression 5.8-, 5.7- and 3.0-fold when incubated in the presence of 2.5 mM theophylline, respectively (Supplementary Table S4). This suggests that all three acted as riboswitches within this organism. It is also worth noting the similarity of the responses by the PC and PC.C63A sequences. This is likely a result of their similar sequence and secondary structure. Meanwhile cells harboring the inactive PC.G81A sequence displayed lower levels of GFP expression. This is consistent with the expectation that this inactive ribozyme has a much lower cleavage rate if any, thereby limiting gene expression. Conversely, cells harboring the constitutively active CA.A52 sequence displayed higher GFP expression levels compared to the PC. This result is line with the expectation that this sequence cleaves at a comparatively faster rate.

One surprising result from Figure 5C is the high normalized GFP expression recorded for the Evo1 construct. For instance, at 2.5 mM theophylline normalized GFP expression reached 1.2 ± 0.1, larger than the remaining constructs, including the constitutively active CA.A52 sequence. To determine whether this result was caused by the Evo1 sequence cleaving at a faster rate, we compared its cleavage response under TRT reaction conditions in the presence of 0 mM and saturating theophylline to that of the PC (Supplementary Figure S8). Although the Evo1 cleavage response was marginally larger in the absence of theophylline, under saturating theophylline its cleavage response was lower, 75.1 ± 0.4 versus 85.95%. This result implies that variations in the 5′ UTR, caused by different ribozyme sequences, affects relative gene expression levels.

## Discussion

The *in vitro* selection of ligand-responsive ribozymes is a powerful approach that enables the identification of rare sequences capable of transducing ligand concentrations into detectable signals. However, the frequency of *in vitro* selection experiments has subsided in recent years due to several limitations.

To reduce the time and human resources required to implement experiments, we developed the LigASERR methodology. We demonstrated the functionality of this approach by enriching a theophylline-responsive ribozyme from a library of 1024 sequences. During this experiment, we demonstrated functional positive and negative selection steps by discriminating against inactive and CA variants, respectively. Importantly, all steps were implemented in a 96-well compatible format, enabling easier scaling compared to previous examples (12, 32). As illustrated in Supplementary Table S3, it is also feasible to conduct positive or negative selections within 8 h and as such a complete selection cycle within two working days. This contrasts with previous methodologies (5, 32) which require more manual manipulation and several overnight incubation steps. It should also be noted that methodologies involving similar processes have previously been automated using liquid-handling robots (33), suggesting full automation can be achieved, reducing time and human resources further.

To optimize selection conditions and to estimate the number of cycles required to enrich the PC, we modeled the process of selection. Investigating a range of selection conditions using the fitness parameter derived from the model, identified TRT reaction conditions as being optimal for the preparation of cDNA. The theoretical work presented here goes beyond previous attempts (34) by providing a framework to predict the dynamics of individual variants during selection. As illustrated during the initial cycles of the Control Experiment, these simulations can be accurate if the majority of sequences are accounted for. It should be possible to generate similarly accurate simulations describing the enrichment of desired sequences from larger and more complicated libraries if the distribution of cleavage responses in these libraries is available. We suspect such parameters can be estimated by measuring the responses of many candidate sequences as has previously been described (6). Estimating these parameters and working within this framework would facilitate a standardized approach to selection, whereby sequence space is systematically explored for the presence of ribozymes with desired functionality.

Following the enrichment of the PC sequence, we tested a modified version of this sequence and two further sequences enriched during selection for their ability to regulate gene expression in *E. coli*. All three induced GFP expression in response to increasing concentrations of theophylline, illustrating their functionality as riboswitches. Furthermore, the 5.7 and 5.8-fold increase in gene expression observed under 2.5 mM theophylline for PC and PC.C63A sequences is similar to the fold increases observed for similar sequences developed using *in vivo* screening methods (7). This illustrates that *in vitro* selection methods can generate riboswitches in *E. coli* with similar functionality to those generated using alternative methods.

We note the PC sequence tested in *E. coli* is equivalent to the previously identified VI-1 sequence. This sequence was only shown to function *in vitro* and had not previously been shown to function as a riboswitch in any cellular environment, failing to regulate gene expression when assayed in mammalian cells (14). There are several potential factors that could explain why the PC sequence failed to function in a mammalian cell environment. One factor is the slower mRNA degradation rates observed in mammalian cells (35, 36). As suggested by other studies (6, 37), a ribozyme with a lower background cleavage rate might yield an effective riboswitch in this cellular environment. Another factor that may explain this result is the presence of different protein actuators required during mammalian mRNA processing (38). Here, we used T7 expression conditions during both selection and when assaying sequences in *E. coli*. Consequently, our *in vitro* expression conditions closely mimicked *in vivo* assays, with a similar rate of transcription and the associated decoupling of transcription from translation observed with T7 (39). The latter ensures more time is available for ribozyme folding prior to perturbations from the translation machinery. The conservation of expression conditions should therefore improve the chances that selected sequences behave in a similar manner when subsequently assayed in *E. coli*.

Based on previous studies (32, 40), upwards of 10^14^ sequences can be sampled using ribozyme *in vitro* selection methods over more than 20 selection cycles. However, compared to previous methods, LigASERR requires an additional transcription, reverse transcription and PCR step per cycle. For all these steps biases exist and while techniques exist to limit these biases, e.g. droplet digital PCR (41), it remains to be determined whether LigASERR can enrich from similar initial pools, over equivalent cycle numbers. Additionally, a complication of *in vitro* selection for riboswitch development as indicated by the results of this work is that factors extrinsic to ribozyme activity can influence absolute levels of gene expression. This was characterized by the higher levels of GFP expression recorded for the Evo1 sequence, even though its measured *in vitro* cleavage rates (Supplementary Figure S8) were similar or lower compared to the PC. As has previously been demonstrated however (42), further work can be implemented to alter gene expression levels by varying factors such as plasmid copy numbers, promoter strengths or perhaps inducer concentrations.

Within this article, we have presented the LigASERR methodology which enables ligand-responsive ribozyme selection at a greater scale, is amenable to automation and requires less time compared to previous methods. We further demonstrated that enriched sequences can function as riboswitches within *E. coli*, highlighting a direct application in small molecule detection and genetic circuit engineering (43). As a final contribution, we presented a model of our selection method which can be used to identify optimal selection conditions and to estimate the dynamics of sequences during selection. We anticipate that the work presented here will form the basis of more ambitious experiments to identify novel ribozyme-based riboswitches, enabling applications involving metabolite detection (44, 45) or therapeutic development (46).

## Supplementary Material

ysz019_Supplementary_Data
