# Supplementary material for: Riboswitch identification using Ligase-Assisted Selection for the Enrichment of Responsive Ribozymes (LigASERR)
Source: Synth Biol (Oxf). 2019 Jul 8;4(1):ysz019. doi: 10.1093/synbio/ysz019 (PMC7445825; doi:10.1093/synbio/ysz019)
Supplement: ysz019_Supplementary_Data [file ysz019_supplementary_data.zip › Haines_et_al_2019_supplementary_data.pdf]

Supplementary Data

Mathematical derivations 2

Fitness of sequence i 2

Assumptions and remarks 2

Derivation 2

Simulated dynamics during selection 6

Assumptions and remarks 6

Derivation 6

Supplementary Material and Methods 9

SPRI beads for cDNA purification 9

SPRI beads for RNA purification 9

Semi-quantitative PCR 9

Supplementary Figures 10

Supplementary Tables 18

References 24

Mathematical derivations

Fitness of sequence i

Assumptions and remarks

The following assumptions were made to derive the expression for fitness in Equation ($S15$):

Assumption 1. There are no non-specific biases during the processes of selection e.g. during transcription, reverse transcription, selection (ligation) or PCR amplification.
Assumption 2. Transcription is assumed to function as a pseudo-first-order chemical reaction with the rate directly proportional to the number of molecules for each individual sequence.
Assumption 3. Individual sequences do not influence the behaviour of one another during selection.

Remark 1. We acknowledge that Assumptions 1 & 2 are unlikely to account for the true process of selection. However, their use avoids having to estimate parameters which would otherwise be difficult to gauge. Furthermore, an ideal selection method would adhere to these assumptions and as such the derivations which follow give an indication to the maximum performance of this system.
Remark 2. It is probable that sequences with regions of complementarity exist within the pool. Such sequences would interact with one another to some degree, potentially affecting Assumption 3. While the magnitude of this affect is unknown, it is likely to be low given the relative concentration of such sequences.

Derivation

To determine the propensity for sequence i to be enriched we model the process of selection as an evolving system, as has previously been described (1). This model states that the number of molecules of sequence i (x_i) after the nth selection ($x_i^{(n+1)}$) is given by:

$$x_i^{(n+1)} = x_i^{(n)}(k_i^{(n)} + 1) \qquad (S1)$$

where $k_i^{(n)}$ is the growth rate of sequence i evaluated at the nth selection. This has previously been interpreted as the number of new individuals each individual produces per selection. If no molecules of sequence i are selected during the nth selection, $k_i^{(n)} = -1$, and sequence i is lost from the selection pool. Otherwise, $k_i^{(n)} > -1$. It should also be noted that selections are discrete, such that $n \in \boldsymbol{Z}_0^+$.

Selection cycles are composed of two selections, positive and negative. To evaluate the performance of sequence i over the course of a cycle, Equation ($S1$) must be modified. Following a cycle, two selections occur, implying the molar amount of sequence i ($x_i^{(n+2)}$) is given by:

$$x_i^{(n+2)} = x_i^{(n)}(k_i^{(n)}k_i^{(n+1)} + k_i^{(n)} + k_i^{(n+1)} + 1) \qquad (S2)$$

Dividing both sides of Equation ($S2$) by $x_i^{(n)}$, yields an expression for the rate of enrichment of sequence i ($\frac{x_i^{(n+2)}}{x_i^{(n)}}$) during a selection cycle.

$$\frac{x_i^{(n+2)}}{x_i^{(n)}} = k_i^{(n)}k_i^{(n+1)} + k_i^{(n)} + k_i^{(n+1)} + 1 \quad (S3)$$

To obtaining a deeper understanding of the factors which affect the rate of enrichment, the growth rate constants in Equation ($S3$) need to be evaluated in terms of parameters and variables which can be measured. To achieve this aim, the mechanics of the selection process was considered (Figure 1, main text). In the ideal case, positive selection should select cleaved cDNA molecules without bias. From this set, some cleaved cDNA molecules will belong to sequence i (C_i). As a result, the molar amount of sequence i after a positive selection ($x_i^{(n^{+}+1)}$) becomes:

$$x_i^{(n^{+}+1)} = \frac{C_i^{(n^{+})}}{\sum_i C_i^{(n^{+})}} \sum_i x_i^{(n)} \quad (S4)$$

The "n^{+}" notation in Equation ($S4$) indicates that the nth selection was a positive selection. This distinction is important considering positive and negative selections differ in at least the concentration of ligand used and the cDNA molecules which are selected for. Conversely, during an ideal negative selection, full-length cDNA molecules should be selected without bias. Again, from this set some will belong to sequence i (F_i), implying:

$$x_i^{(n^{-}+1)} = \frac{F_i^{(n^{-})}}{\sum_i F_i^{(n^{-})}} \sum_i x_i^{(n)} \quad (S5)$$

where $x_i^{(n^{-}+1)}$ is the number of molecules of x_i after the nth round of selection, given negative selection was implemented (n^{-}).

Equations ($S4$) & ($S5$) were substituted into Equation ($S1$) to derive expressions for the growth rate constants during positive ($k_i^{(n^{+})}$) and negative ($k_i^{(n^{-})}$) selection. With further algebraic manipulation, this yielded Equations ($S6$) and ($S7$), respectively.

$$k_i^{(n^{+})} = \left(\left(\frac{C_i^{(n^{+})}}{x_i^{(n)}} \right) \div \left(\frac{\sum_i C_i^{(n^{+})}}{\sum_i x_i^{(n)}} \right) \right) - 1 \quad (S6)$$

$$k_i^{(n^{-})} = \left(\left(\frac{F_i^{(n^{-})}}{x_i^{(n)}} \right) \div \left(\frac{\sum_i F_i^{(n^{-})}}{\sum_i x_i^{(n)}} \right) \right) - 1 \quad (S7)$$

Without simplifying the expressions in Equations ($S6$) and ($S7$) further, any measure of the rate of enrichment would be dependent on $x_i^{(n)}$. To avoid having to calculate this variable, we use Assumptions 1 & 2, writing $C_i^{(n)} + F_i^{(n)}$ as:

$$C_i^{(n)} + F_i^{(n)} = k_T t k_{RT} x_i^{(n)}$$

Where t is the length of time transcription is incubated for, k_T is the transcription reaction rate constant and k_{RT} is the efficiency of reverse transcription. Rearranging this expression to solve for $x_i^{(n)}$ yields:

$$x_i^{(n)} = \frac{C_i^{(n)} + F_i^{(n)}}{k_T t k_{RT}} \qquad (S8)$$

Using Equation $(S8)$, $x_i^{(n)}$ and $\sum_i x_i^{(n)}$ in Equations $(S6)$ and $(S7)$ can be substituted. With additional algebraic manipulation, Equations $(S9)$ and $(S10)$ are evaluated, respectively.

$$k_i^{(n^+)} = \left(\left(\frac{C_i^{(n^+)}}{F_i^{(n^+)} + C_i^{(n^+)}}\right) \div \left(\frac{\sum_i C_i^{(n^+)}}{\sum_i \left(C_i^{(n^+)} + F_i^{(n^+)}\right)}\right)\right) - 1 \qquad (S9)$$

$$k_i^{(n^-)} = \left(\left(1 - \frac{C_i^{(n^-)}}{F_i^{(n^-)} + C_i^{(n^-)}}\right) \div \left(1 - \frac{\sum_i C_i^{(n^-)}}{\sum_i \left(C_i^{(n^-)} + F_i^{(n^-)}\right)}\right)\right) - 1 \qquad (S10)$$

The terms $\frac{C_i^{(n^+)}}{F_i^{(n^+)} + C_i^{(n^+)}}$ and $\frac{C_i^{(n^-)}}{F_i^{(n^-)} + C_i^{(n^-)}}$ are equivalent to the cleaved fraction of sequence i under positive and negative selection conditions, respectively. Under Assumption 3 these terms will not vary (2) and as such are independent of n. With this realisation Equations $(S9)$ and $(S10)$ are rewritten as:

$$k_i^{(n^+)} = \frac{r_i^{(+)}}{R^{(n^+)}} - 1 \qquad (S11)$$

$$k_i^{(n^-)} = \frac{1 - r_i^{(-)}}{1 - R^{(n^-)}} - 1 \qquad (S12)$$

Where $r_i^{(+)}$ and $r_i^{(-)}$ are defined as the fraction of sequence i which is cleaved under positive and negative selection conditions, respectively. In addition to being constant parameters, $r_i^{(+)}$ and $r_i^{(-)}$ can be calculated empirically as demonstrated by Figure 3 and Figure S2. The remaining variables, $R^{(n^+)}$ and $R^{(n^-)}$ are given below in Equations $(S13)$ and $(S14)$:

$$R^{(n^+)} = \frac{\sum_i C_i^{(n^+)}}{\sum_i \left(C_i^{(n^+)} + F_i^{(n^+)}\right)} \qquad (S13)$$

$$R^{(n^-)} = \frac{\sum_i C_i^{(n^-)}}{\sum_i \left(C_i^{(n^-)} + F_i^{(n^-)}\right)} \qquad (S14)$$

To understand how the parameters $r_i^{(+)}$ and $r_i^{(-)}$ affect the rate of enrichment for sequence i, $k_i^{(n)}$ and $k_i^{(n+1)}$ from Equation $(S3)$ are substituted with $k_i^{(n^+)}$ and $k_i^{(n^-)}$ from Equations $(S11)$ and $(S12)$. With additional manipulation this yields a variation of Equation (1) in the main text:

$$R^{(n^+)}\left(1 - R^{(n^-)}\right)\frac{x_i^{(n+2)}}{x_i^{(n)}} = r_i^{(+)}\left(1 - r_i^{(-)}\right) \equiv fitness_i \tag{S15}$$

Equation ($S15$) illustrates how an expression involving the parameters, $r_i^{(+)}$ and $r_i^{(-)}$ is directly proportional with the rate of enrichment for sequence i ($\frac{x_i^{(n+2)}}{x_i^{(n)}}$). We refer to this expression as the fitness of sequence i ($fitness_i$). We note that while technically either $R^{(n^+)}$ or $R^{(n^-)}$ in Equation ($S15$) must be evaluated at the $(n+1)^{th}$ selection, this issue only needs to be addressed if the relationship between fitness and the rate of enrichment requires quantification. This issue can be avoided by dividing the fitness values of two sequences. Given that this term applies equally to all sequences, it cancels upon division. This implies that the selection conditions which maximise the enrichment of one sequence over another can be determine using only the fitness's of the two sequences under consideration.

Simulated dynamics during selection

Assumptions and remarks

In addition to the assumptions made to derive the expression in Equation ($S15$), the following assumptions were made in order to derive the expressions in Equations ($S22$) & ($S23$):

Assumption 4. All sequences in the starting library are present at the same initial frequency.
Assumption 5. The total number of molecules within the pool does not vary between selections.
Assumption 6. For the purposes of calculating the dynamics of a single sequence (i), all remaining sequences are assumed to have identical dynamics during selection.

Remark 3. Although biases in DNA synthesis will affect Assumption 4, this approximation is often used when gauging library coverage.
Remark 4. Given the use of semi-quantitative PCR to amplify the pool following selection, Assumption 5 is likely to be accurate.
Remark 5. Limitations and potential improvements to Assumption 6 are discussed within the Results & Discussion sections of the main text.

Derivation

The dynamics of sequence i during selection can be summarised by its initial frequency ($\frac{x_i^{(0)}}{\sum_i x_i^{(0)}}$) and its frequency following each subsequent selection, equivalent to:

$$\left[\frac{x_i^{(0)}}{\sum_i x_i^{(0)}}, \frac{x_i^{(1)}}{\sum_i x_i^{(1)}}, \frac{x_i^{(2)}}{\sum_i x_i^{(2)}} \dots, \frac{x_i^{(N)}}{\sum_i x_i^{(N)}}\right],$$

Where N is the total number of selections implemented. To calculate this vector, it is necessary to know the initial frequency of sequence i and a general method for calculating the frequency of sequence i following a selection ($\frac{x_i^{(n+1)}}{\sum_i x_i^{(n+1)}}$).

Under Assumption 4, $\frac{x_i^{(0)}}{\sum_i x_i^{(0)}}$ can be calculated from the number of sequences in the starting library (I):

$$\frac{x_i^{(0)}}{\sum_i x_i^{(0)}} = \frac{1}{I} \qquad (S16)$$

In calculating $\frac{x_i^{(n+1)}}{\sum_i x_i^{(n+1)}}$, two expressions must be generated. This is because different frequencies would be generated depending on whether positive or negative selection takes place. To calculate an expression for the frequency of sequence i following positive selection ($\frac{x_i^{(n^{+}+1)}}{\sum_i x_i^{(n^{+}+1)}}$), $k_i^{(n^{+})}$ from Equation

$(S11)$ is substituted into Equation $(S1)$ and the resulting expression divided by the total number of molecules. Under Assumption 5 ($\sum_i x_i^{(n^+ + 1)} \equiv \sum_i x_i^{(n)}$) this yields:

$$\frac{x_i^{(n^+ + 1)}}{\sum_i x_i^{(n^+ + 1)}} = \left(\frac{x_i^{(n)}}{\sum_i x_i^{(n)}}\right)\left(\frac{r_i^{(+)}}{R^{(n^+)}}\right) \tag{S17}$$

The presence of $R^{(n^+)}$ in Equation $(S17)$ implies that this expression cannot be calculated without knowledge regarding the performance of the pool during the nth selection. The remainder of this derivation addresses this issue so that $\frac{x_i^{(n^+ + 1)}}{\sum_i x_i^{(n^+ + 1)}}$ can be calculated without considering this variable.

Our first step was to substitute the numerator of Equation $(S13)$, leading $R^{(n^+)}$ to be written in terms of $r_i^{(+)}$:

$$R^{(n^+)} = \frac{\sum_i \left(r_i^{(+)}\left(C_i^{(n^+)} + F_i^{(n^+)}\right)\right)}{\sum_i \left(C_i^{(n^+)} + F_i^{(n^+)}\right)} \tag{S18}$$

Using the expression in Equation $(S8)$, the "$C_i^{(n^+)} + F_i^{(n^+)}$" terms in Equation $(S18)$ can be substituted. With further manipulation this yields:

$$R^{(n^+)} = \sum_i \left(\frac{x_i^{(n)}}{\sum_i x_i^{(n)}} r_i^{(+)}\right) \tag{S19}$$

Equation $(S19)$ implies that if the frequency along with the fraction cleaved responses for each sequence is known, $R^{(n^+)}$ can be calculated. Although the initial frequency of each sequence can be estimated initially using Equation $(S16)$, measuring $r_i^{(+)}$ for all sequences (1 through I) is not feasible. To overcome this issue, Assumption 6 is used. So that sequences other than sequence i have identical dynamics during selection, we represent their fraction cleaved response as a single parameter ($r_j^{(+)}$), implying:

$$R^{(n^+)} = \frac{x_i^{(n)}}{\sum_i x_i^{(n)}} r_i^{(+)} + \left(1 - \frac{x_i^{(n)}}{\sum_i x_i^{(n)}}\right) r_j^{(+)} \tag{S20}$$

To solve for $r_j^{(+)}$, we use $\frac{x_i^{(n)}}{\sum_i x_i^{(n)}}$ and $R^{(n^+)}$ values calculated at the 0th round, prior to selection. With the identity given in Equation $(S16)$, $r_j^{(+)}$ is evaluated to:

$$r_j^{(+)} = \frac{IR^{(0^+)} - r_i^{(+)}}{I - 1} \tag{S21}$$

By substituting Equation $(S21)$ into Equation $(S20)$ and the resulting expression into Equation $(S17)$, an expression for $\frac{x_i^{(n^+ + 1)}}{\sum_i x_i^{(n^+ + 1)}}$ is yielded:

$$\frac{x_i^{(n^{+}+1)}}{\sum_i x_i^{(n^{+}+1)}} = \frac{\frac{x_i^{(n)}}{\sum_i x_i^{(n)}} r_i^{(+)}}{\frac{x_i^{(n)}}{\sum_i x_i^{(n)}} r_i^{(+)} + \left(1 - \frac{x_i^{(n)}}{\sum_i x_i^{(n)}}\right) \frac{IR^{(0^{+})} - r_i^{(+)}}{I-1}} \tag{S22}$$

Repeating this process to calculate the frequency of sequence i following a negative selection ($\frac{\mathrm{x}_{\mathrm{i}}^{(\mathrm{n}^{-}+1)}}{\sum_i x_i^{(n^{-}+1)}}$), yields Equation ($S23$):

$$\frac{x_i^{(n^{-}+1)}}{\sum_i x_i^{(n^{-}+1)}} = \frac{\frac{x_i^{(n)}}{\sum_i x_i^{(n)}} \left(1 - r_i^{(-)}\right)}{1 - \left(\frac{x_i^{(n)}}{\sum_i x_i^{(n)}} r_i^{(-)} + \left(1 - \frac{x_i^{(n)}}{X}\right) \frac{I\,R^{(0^{-})} - r_i^{(-)}}{I-1}\right)} \tag{S23}$$

Equations ($S16$), ($S22$) and ($S23$) imply that the dynamics of sequence i can be estimated so long as the order of positive and negative selections are known, along with the following parameters: I, $r_i^{(+)}$, $r_i^{(-)}$, $R^{(0^{+})}$ and $\mathrm{R}^{(0^{-})}$. Importantly, all these parameters can be measured prior to selection.

Supplementary Material and Methods

SPRI beads for cDNA purification

Agencourt AMPure XP beads (Beckman Coulter) were resuspended in an equivalent volume of 10 mM Tris-HCl (pH 8.0), 1 mM EDTA, 39 % (w/v) PEG 8000, 0.05 % Tween 20 and either 0.445 or 1 M NaCl, depending on whether samples were previously washed with Dynabeads or not. Similar to previous efforts (3), beads were pelleted and washed twice with 1.5x the volume of 10 mM Tris-HCl (pH 8.0), 1 mM EDTA, 0.05 % Tween 20 buffer before being resuspended in a buffer devoid of PEG 8000 & Tween 20. These two latter components were subsequently added with vigorous mixing and/or vortexing.

1.8x volume of SPRI beads was added to samples and the remaining protocol outlined by the manufacturer followed, eluting in nuclease-free water.

SPRI beads for RNA purification

SPRI beads for RNA purification were generated and utilised in an equivalent manner to that described above for cDNA purification, except 10 mM Tris-HCl (pH 8.0) in buffers was substituted with 1 mM Sodium citrate, pH 6.4.

Semi-quantitative PCR

The following semi-quantitative PCR workflow was inspired by the work of Klussmann and colleagues (4). Semi-quantitative PCRs were amplified using PFU DNA polymerase (Promega) and 0.5 µM Sense_bio and Anti-sense_bio primers. PCRs were incubated for 10 cycles with a final extension of 2 minutes. PCRs were then held at 50 °C and 20 µL off-sampled and DNA quantified using a Qubit® Fluorometer 2.0 and dsDNA BR Assay kit (Thermo Fisher Scientific). If the concentration of DNA (X) exceeded 2.24 ng/µL, the remaining number of cycles (C) required to achieve a yield as close to 25.1 ng/µL as possible was calculated using the expression below:

$$C \leq \log(\frac{25.1}{X})/\ \log(1.65)$$

If the concentration of DNA was below 2.24 ng/µL, a further 5 cycles was conducted and the concentration of DNA remeasured. No more than 16 PCR cycles were implemented during a selection.

Supplementary Figures

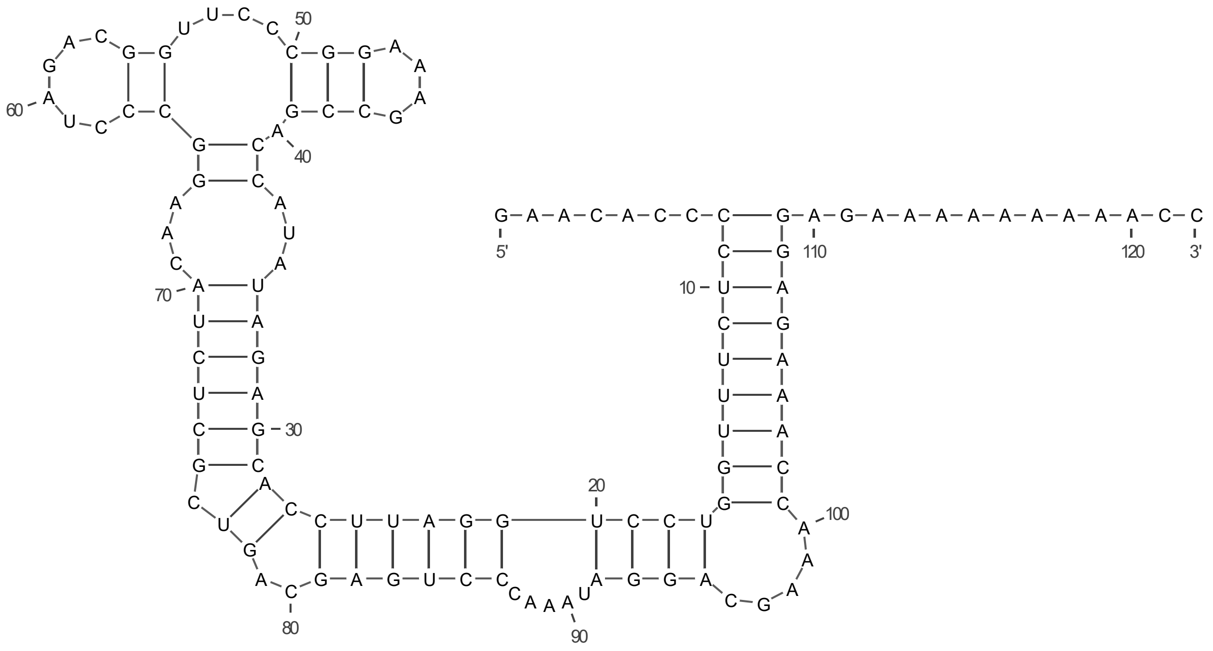

Figure S1. Predicted, Positive Control full-length RNA minimal free energy secondary structure.

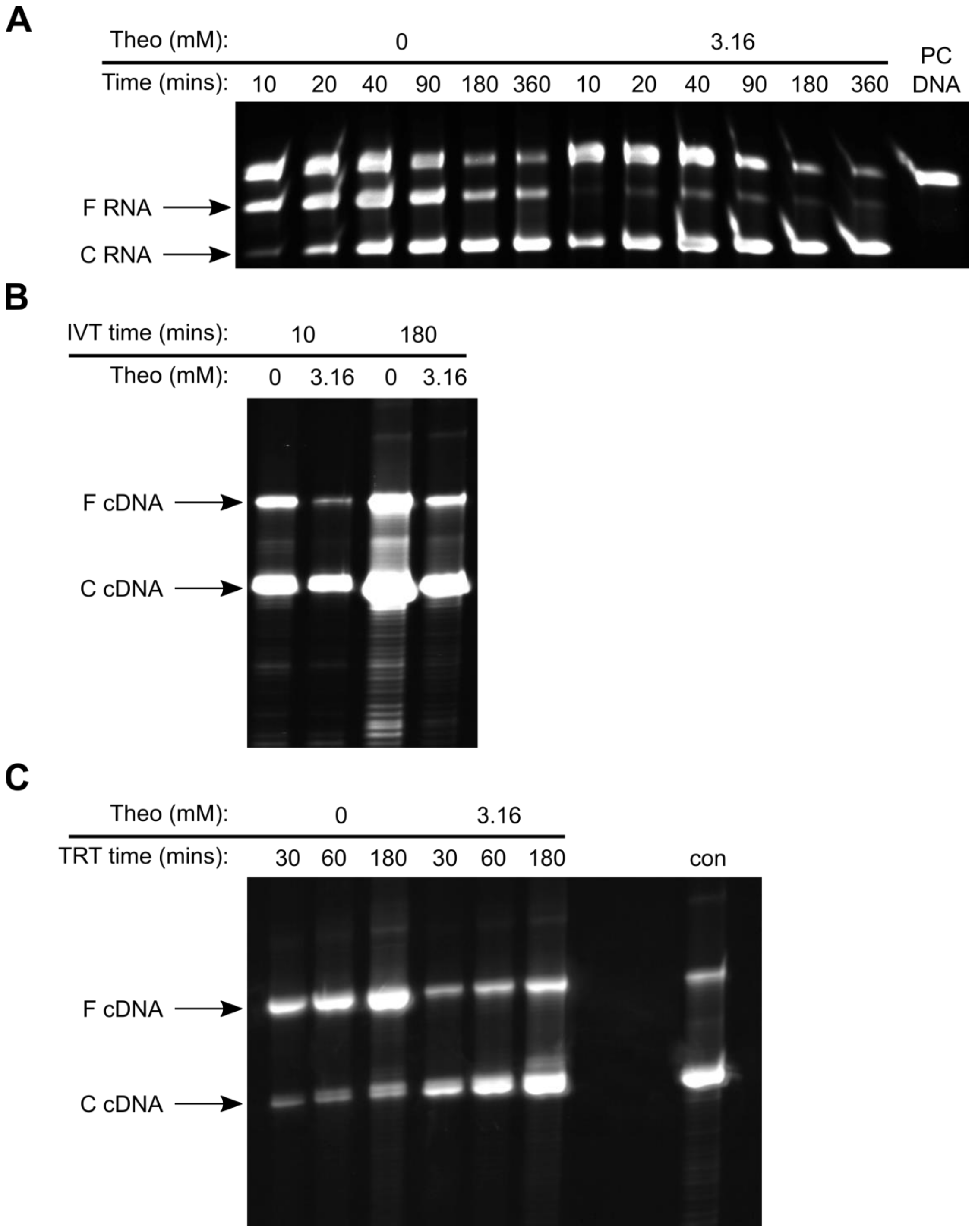

Figure S2. Raw data for Figure 3 (main text). *In vitro* transcription (IVT), reverse transcription and TRT reactions were incubated under 0 or 3.16 mM theophylline (Theo) for varying times as described in the "Materials and methods – *In vitro* ribozyme assays" section. (A) Positive Control (PC) RNA with DNA loaded for comparison. Similar quantities of RNA were loaded. (B) PC cDNA synthesised from purified RNA, generated with varying IVT times. (C) PC cDNA synthesised under TRT reaction conditions. The sample from (B), generated under 0 mM theo and *in vitro* transcribed for 180-minutes, is loaded for comparison (con).

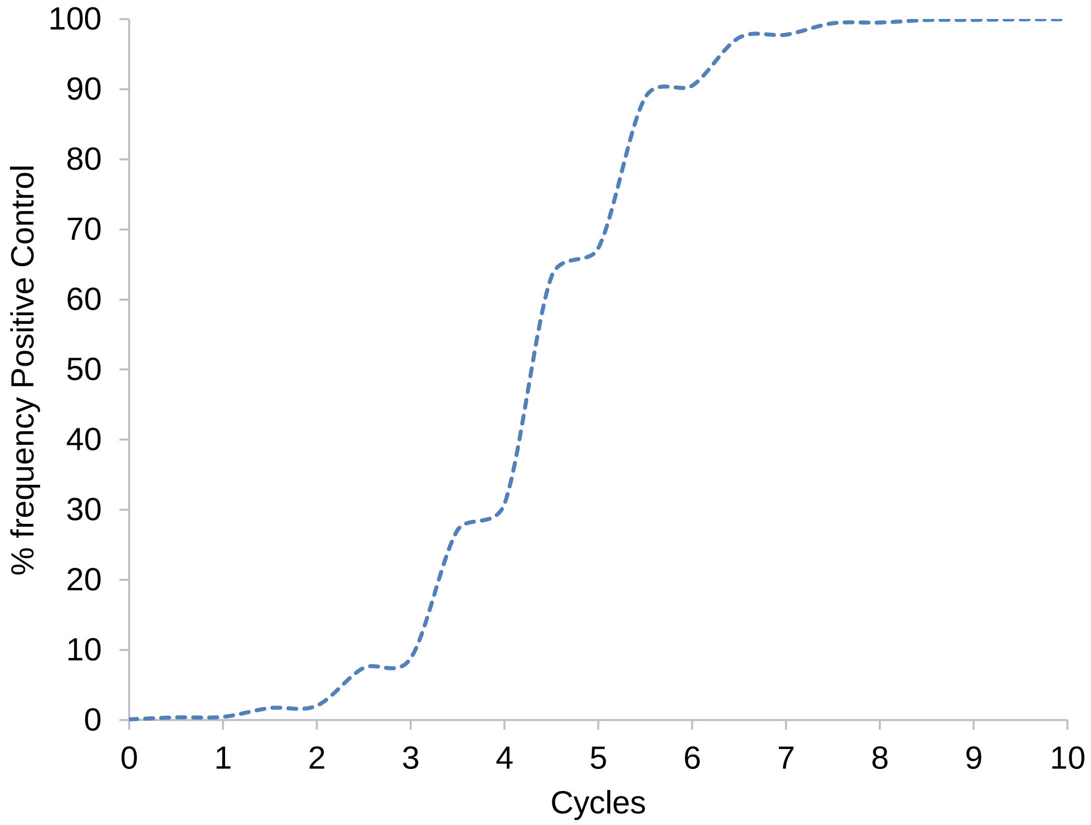

Figure S3. Predicted enrichment of the Positive Control sequence from the Control Library. The initial frequency of the Positive Control sequence was calculated according to Equation $(S16)$. Alternating positive/negative selection cycles were implemented starting with a positive selection, using Equations $(S22)$ & $(S23)$, along with the parameters given in Table S2.

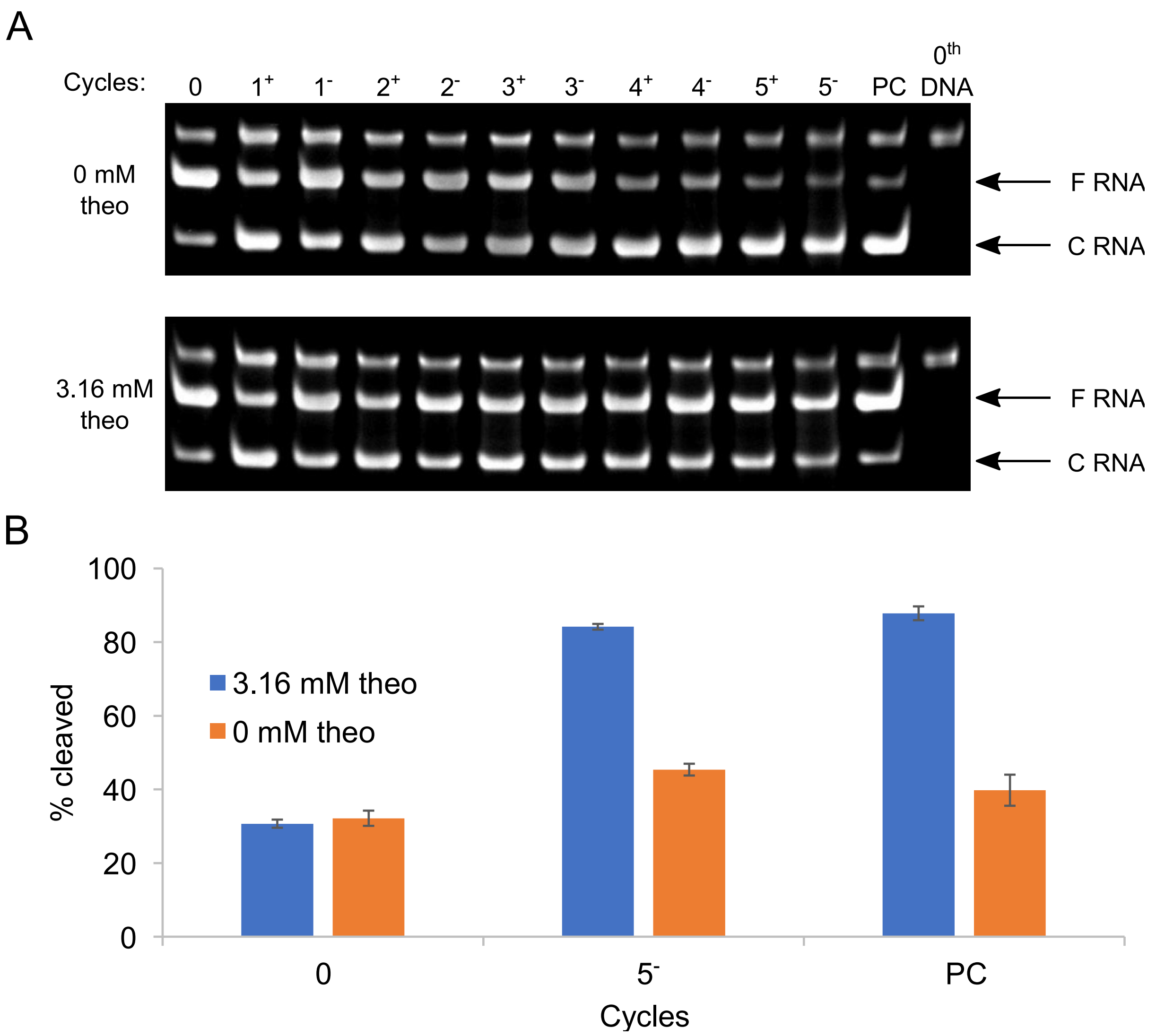

Figure S4. Enrichment of a theophylline-activated phenotype from the Control Library. (A) The pool following each selection was *in vitro* transcribed in 0 (top) and 3.16 (bottom) mM theophylline. The Positive Control (PC) and DNA from the initial pool (0th) are loaded for comparison. Full-length (F) and cleaved (C) RNA are indicated. (B) % cleaved values calculated from the pool after the 0th or 5^{-th} cycles of selection are illustrated along with values for the Positive Control. Error bars indicate SDs from 4 repeats.

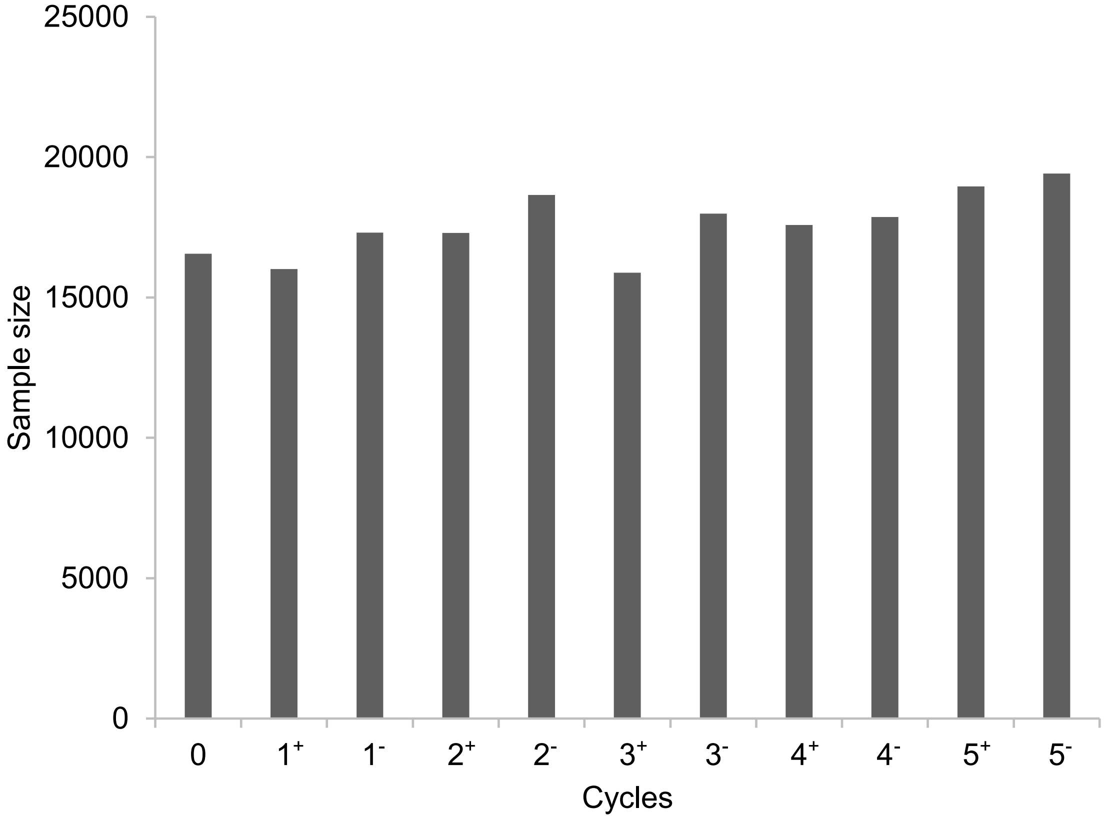

Figure S5. Sample size of the selection pool as a function of selection cycles. The sample size is equivalent to the number of trimmed and merged pair-end reads generated through NGS.

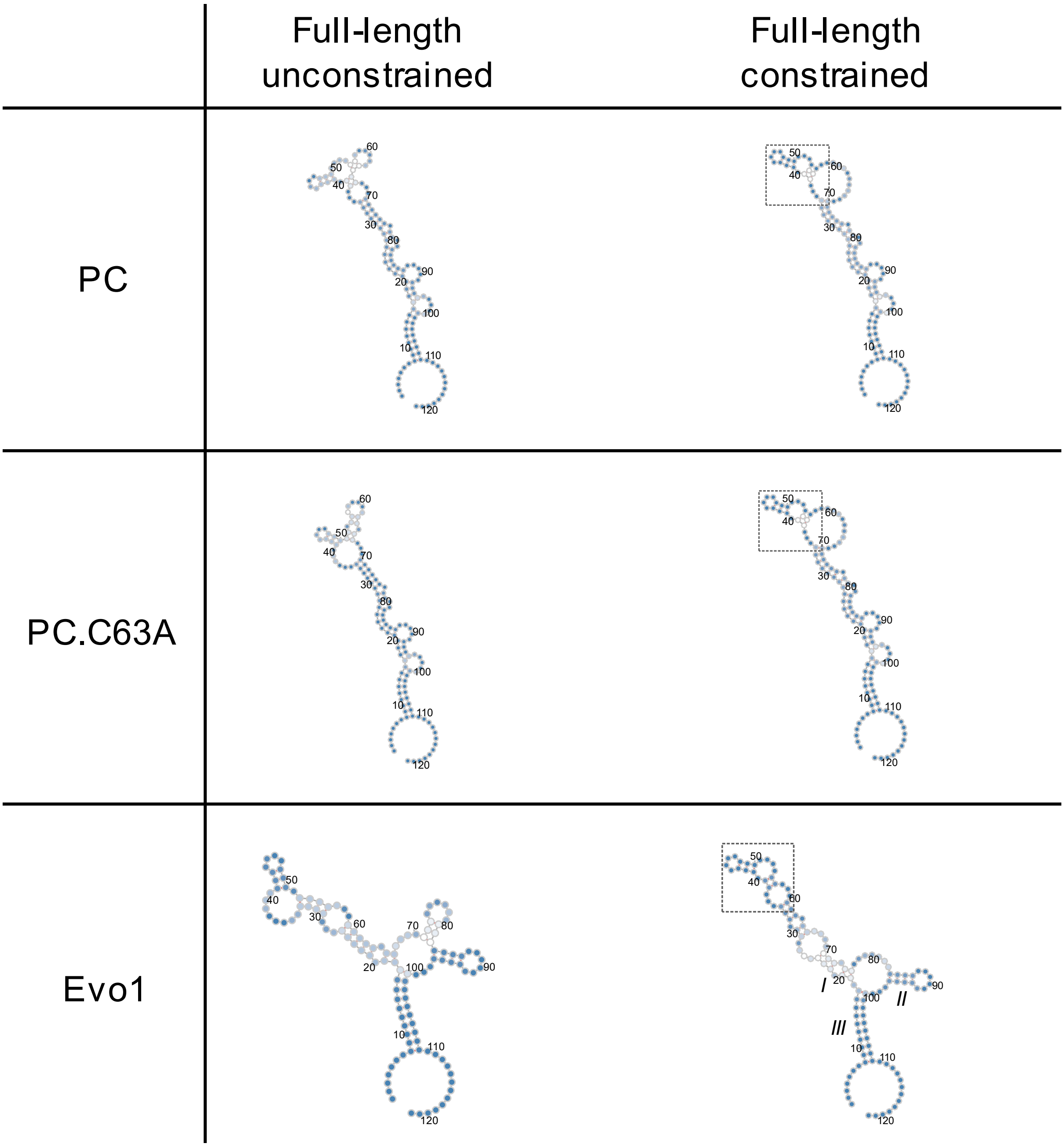

Figure S6. Predicted minimum free energy secondary structures for enriched theophylline-ribozyme sequences. Full-length Positive Control (PC), PC.C63A and Evo1 sequences were folded with and without constrained theophylline aptamer domains (grey-dotted line). Colouring indicates base-pair probabilities with white and blue indicating low and high probabilities, respectively. Sequences are numbered 5' to 3'. For the constrained Evo1 sequence, hammerhead ribozyme stems I, II and III are indicated.

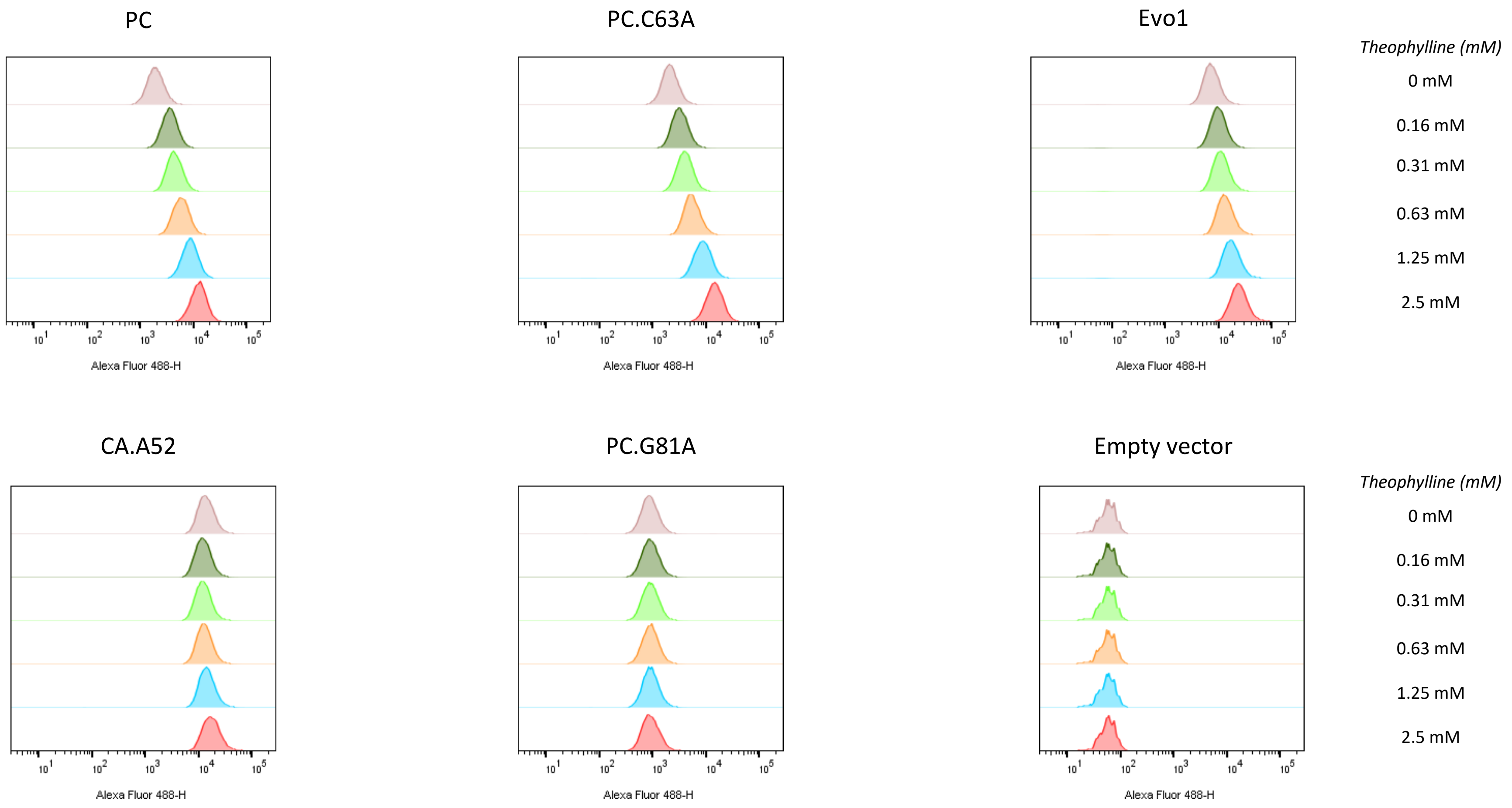

Figure S7. Flow cytometry histograms for one of two data sets used to generate the graph in Figure 5 (main text).

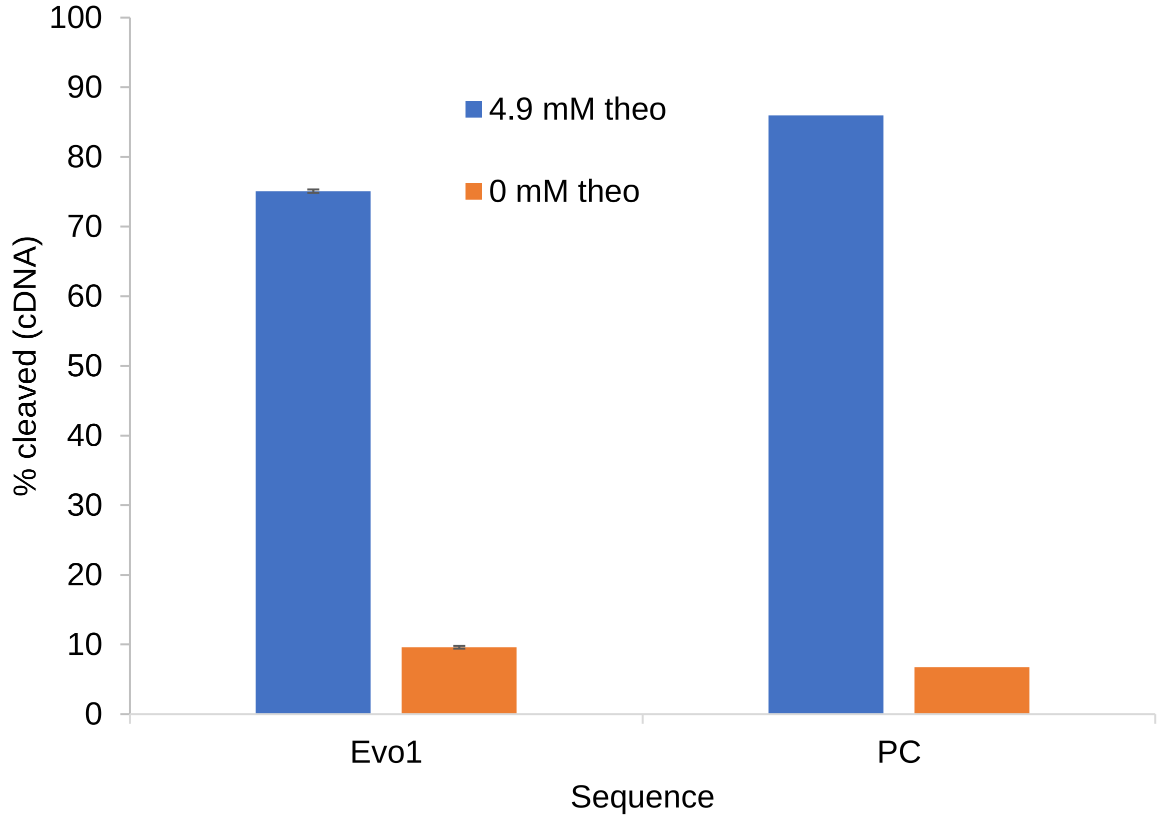

Figure S8. *In vitro* responses of Evo1 and PC sequences. DNA templates were incubated under TRT reaction conditions in the presence of 4.9 or 0 mM theophylline and the resulting cDNA separated via electrophoresis for analysis of cleavage (Materials and methods). For the Evo1 sequence, error bars denote standard deviations from 3 repeats.

Supplementary Tables

Sequence	Positive selection	Negative selection
Positive Control	C >> F (↑)	F >> C (↑)
Inactive	F >> C (↓)	F >> C (↑)
Constitutively active	C >> F (↑)	C >> F (↓)

Table S1. Behaviour and dynamics of sequences during positive and negative selection. The "C >> F" notation indicates more cleaved than full-length cDNA is expected under the corresponding selection conditions. The "F >> C" notation indicates the opposite. The parenthesised up or down arrow indicates an expected increase or decrease in frequency, respectively, following the corresponding selection.

Parameter	Value	Reasoning
$R^{(0^{+})}$	0.21	Measured Control Library cleavage response under 3.16 mM theo
$R^{(0^{-})}$	0.21	Measured Control Library cleavage response under 0 mM theo
$r_i^{(+)}$	0.81	Measured Positive Control cleavage response under 3.16 mM theo
$r_i^{(-)}$	0.05	Measured Positive Control cleavage response under 0 mM theo
I	1024	Number of possible sequences in the Control Library

Table S2. Parameters used to simulate the selection dynamics of the Positive Control given to 2 decimal places (Figure S3 and in Figure 4A in the main text).

Step	Time (minutes)
Prepare TRT reaction	30
Incubate TRT reaction	50
Non-specific cDNA purification	135
Ligation of selected cDNA using *Tth* DNA Ligase	90
Pull-down & purification of ligation reaction products	50
Semi-qPCR	60
PCR purification	50
Total (hours)	7.75

Table S3. Time required to implement LigASERR. Times are accurate for 8 samples conducted in parallel.

Sequence	Geometric mean (0 mM)	Geometric mean (2.5 mM)	Fold increase
PC	1880	10848	5.8
PC.C63A	2117	12038.5	5.7
Evo1	6587	19616.5	3.0
CA.A52	13208.5	16003	1.2
PC.G81A	828	902.5	1.1
Empty vector	54.95	54.75	1.0

Table S4. Absolute GFP fluorescence for cells harbouring selected sequences. Geometric mean fluorescence (n = 2) at specified theophylline concentrations was evaluated from flow cytometry data (Figure S7).

ID	Sequence	IDT purification code
Adapter F	/5Phos/TATAGTGAGTCGTATTA/3Bio/	HPLC
Adapter C	/5Phos/GACCAAAGAGGGGTGTTCTATAGTGAGTCGTATTA/3Bio/	HPLC
Anti-sense	GGTTTTTTTTTCTCCTCTTTGGTTTCGTCCTA	STD
Anti-sense_bio	/5Biosg/GGTTTTTTTTTCTCCTCTTTGGTTTCGTCCTA	STD
MH_IllumAnti	GTCTCGTGGGCTCGGAGATGTGTATAAGAGACAGGGTTTTTTTTTCTCCTCTTTGGTTTCGTCCTA	STD
MH_IllumSense	TCGTCGGCAGCGTCAGATGTGTATAAGAGACAGTAATACGACTCACTATAGAACACCCCTCTTTG	STD
RT primer	GGTTTTTTTTTCT	STD
RT primer (HEX-labelled)	/5HEX/GGTTTTTTTTTCT	HPLC
Sense	TAATACGACTCACTATAGAACACCCCTCTTTGGTCCTGGATTC	STD
Sense_bio	/5Biosg/TAATACGACTCACTATAGAACACCCCTCTTTGGTCCTGGATTC	STD
Evo1	TAATACGACTCACTATAGAACACCCCTCTTTGGTCCTGGATTCCACGAGATATACCAGCCGAAAGGCCCTTGGCAGATCCCGGATCATCGAGCTGACGAGTCCCAAATAGGACGAAACCAAAGAGGAGAAAAAAAAACC	STD
Splint	TAATACGACTCACTATAGAACACCCCTCTTTGGTCCTGGATTCCAC/3C6/	HPLC
Positive Control	TAATACGACTCACTATAGAACACCCCTCTTTGGTCCTGGATTCCACGAGATATACCAGCCGAAAGGCCCTTGGCAGATCCCGGAACATCTCGCTGACGAGTCCCAAATAGGACGAAACCAAAGAGGAGAAAAAAAAACC	STD
Control Library	TAATACGACTCACTATAGAACACCCCTCTTTGGTCCTGGATTCCACGAGATATACCAGCCGAAAGGCCNTTGGCAGATCNCGGAACATCNNGCTGACNAGTCCCAAATAGGACGAAACCAAAGAGGAGAAAAAAAAACC	STD
SEVA_T0_rev	GGACCCCTGGATTCTCACC	STD

SEVA_T1_for	GGCGGCGGATTTGTCCTAC	STD

Table S5. Chemically synthesised oligonucleotides acquired from Integrated DNA Technologies, Inc. – The IDT purification code for each sequence is given. “/5Phos/”, “/5HEX/”, “/5Biosg/” & “/3C6/” denote the presence of a 5’ Phosphate group, 5’ HEX, 5’ standard biotin or 3' Hexanediol group, respectively. In all sequences the T7 promoter is highlighted in magenta. Sequences within the splint highlighted in green anneal to cleaved cDNA in the presence of Adapter C. Sequences within the splint apart from the T7 promoter anneal to full-length cDNA in the presence of Adapter F. Table S5 is additionally available in csv format online.

References

1. Taylor,P.D. and Jonker,L.B. (1978) Evolutionary stable strategies and game dynamics. *Math. Biosci.*, **40**, 145–156.

2. Long,D.M. and Uhlenbeck,O.C. (1994) Kinetic characterization of intramolecular and intermolecular hammerhead RNAs with stem II deletions. **91**, 6977–6981.

3. Clarke,A.C., Prost,S., Stanton,J.-A.L., White,W.T.J., Kaplan,M.E., Matisoo-Smith,E.A. and Genographic Consortium (2014) From cheek swabs to consensus sequences: an A to Z protocol for high-throughput DNA sequencing of complete human mitochondrial genomes. *BMC Genomics*, **15**, 68.

4. Eulberg,D., Buchner,K., Maasch,C. and Klussmann,S. (2005) Development of an automated in vitro selection protocol to obtain RNA-based aptamers: identification of a biostable substance P antagonist. *Nucleic Acids Res.*, **33**, e45.
